# Associations between Long-Term Particulate Matter Exposure and Adult Renal Function in the Taipei Metropolis

**DOI:** 10.1289/EHP302

**Published:** 2016-10-07

**Authors:** Ya-Ru Yang, Yung-Ming Chen, Szu-Ying Chen, Chang-Chuan Chan

**Affiliations:** 1 Institute of Occupational Medicine and Industrial Hygiene, College of Public Health, National Taiwan University, Taipei, Taiwan; 2 Department of Internal Medicine, National Taiwan University Hospital, Taipei, Taiwan; 3 Division of Surgical Intensive Care, Department of Critical Care Medicine, E-Da Hospital, Kaohsiung, Taiwan

## Abstract

**Background:**

Studies on the effect of air pollutions on kidney diseases are still limited.

**Objective:**

We aimed to investigate the associations between particulate matter (PM) exposures and renal function among adults.

**Methods:**

We recruited 21,656 adults as participants from 2007 to 2009. The Taiwanese Chronic Kidney Disease Epidemiology Collaboration (CKD-EPI) equation was used to derive the estimated glomerular filtration rate (eGFR). Subjects with an eGFR lower than 60 mL/min/1.73 m^2^ were defined as having chronic kidney disease (CKD). Land use regression (LUR) models were used to estimate individual exposures to PM with an aerodynamic diameter < 10 μm (PM_10_), coarse particles (PM_Coarse_), fine particles (PM_2.5_), and PM_2.5Absorbance_. Generalized linear and logistic regression models were used to estimate the associations between PM exposure and renal function.

**Results:**

An IQR increase in PM_10_ (5.83 μg/m^3^) was negatively associated with eGFR by –0.69 (95% CI: –0.89, –0.48) mL/min/1.73 m^2^ and positively associated with the prevalence of CKD with adjusted OR = 1.15 (95% CI: 1.07, 1.23). An IQR increase in PM_Coarse_ (6.59 μg/m^3^) was significantly associated with lower eGFR by –1.07 (95% CI: –1.32, –0.81) mL/min/1.73 m^2^ and CKD with OR = 1.26 (95% CI: 1.15, 1.38). In contrast, neither outcome was significantly associated with PM_2.5_ or PM_2.5Absorbance_. Stratified analyses indicated that associations of CKD with both PM_10_ and PM_Coarse_ were limited to participants < 65 years of age, and were stronger (for PM_10_) or limited to (PM_Coarse_) women. Associations also appeared to be stronger in those without (vs. with) hypertension, and in normal versus overweight participants.

**Conclusions:**

Exposure during the previous year to PM_10_ and PM_Coarse_, but not PM_2.5_ or PM_2.5Absorbance_, was associated with reduced renal function among Taiwanese adults.

**Citation:**

Yang YR, Chen YM, Chen SY, Chan CC. 2017. Associations between long-term particulate matter exposure and adult renal function in the Taipei metropolis. Environ Health Perspect 125:602–607; http://dx.doi.org/10.1289/EHP302

## Introduction

A large body of evidence supports adverse effects of ambient air pollution on vital organs, including the heart, lungs, and central nervous system (Kampa and Castanas 2008). Recent studies suggest that particulate matter (PM) exerts detrimental effects on cardiovascular outcomes through the induction of oxidative injury and proinflammatory pathways and the resultant development and progression of atherosclerosis (Brook et al. 2010; Mills et al. 2009). The presence of cardiovascular disease (CVD) is known to predict a faster progression of chronic kidney disease (CKD) toward end-stage renal disease (ESRD) (Levin 2003), while patients with CKD have a high risk of developing cardiovascular events and all-cause mortality (Go et al. 2004; Weiner et al. 2004). Because CKD is regarded as a coronary heart disease risk factor (Briasoulis and Bakris 2013; Tonelli et al. 2012), it is tempting to speculate that ambient air pollution or PM may affect kidney function through direct or indirect mechanisms similar to those proposed for cardiovascular effects of these exposures. In Taiwan, ambient air pollutants and PM have been associated with hospital admissions for CVD (Chang et al. 2005; Chen et al. 2015; Hsieh et al. 2010; Yang 2008). In contrast, we are aware of only three observational studies that have explored the potential effects of air pollutants on renal function, with conflicting results (Lue et al. 2013; Mehta et al. 2016; O’Neill et al. 2008). A recent experimental study reported that 16 weeks of exposure to concentrated ambient particles (average 13.3 μg/m^3^) versus filtered air was positively associated with glomerulosclerosis in a rat model of type 1 diabetes (Yan et al. 2014). A recent longitudinal analysis of participants in the VA Normative Aging Study indicated a negative association between PM_2.5_ exposure during the previous year and renal function in older men (Mehta et al. 2016). In 2013, Taiwan was ranked as the country with the highest incidence of ESRD among dozens of countries surveyed (U.S. Renal Data System 2016). Therefore, we conducted a cross-sectional population-based study to investigate associations between particulate matter exposure during the previous year [estimated using a land use regression (LUR) model] and renal function and CKD prevalence among adults residing in the metropolitan area of New Taipei City, Taiwan.

## Materials and Methods

### Study Population

Subjects for the present study were selected from participants in the New Taipei City Health Screening Program, an annual health screening program supervised by the Department of Health of the New Taipei City Government (http://www.health.ntpc.gov.tw/). All citizens > 30 years of age who resided in New Taipei City, Taiwan, were invited to participate in the program every 3 years. The present study included those who participated during 2007–2009 and were residents of the metropolitan area (population density > 20,000/km^2^ in 2009) and who were 30–97 years of age. After excluding 2,630 participants with incomplete information, the final population for the present study included 21,656 participants living in six districts. The Department of Health of the New Taipei City Government approved our use of de-identified screening program data (with names and IDs unlinked from medical records to ensure confidentiality), and the study was approved by the Joint Institutional Review Boards of National Health Research Institutes.

### Health Data

The health screening program conducted by the Department of Health of the New Taipei City Government included a clinician interview, self-reported questionnaire, and collection of a venous blood sample. The clinician interview and questionnaire gathered information that included each subject’s home address, age, sex, height, weight, body mass index (BMI), smoking status, alcohol consumption, and betel nut–chewing status. Measurements of blood pressure were taken once with the subject seated in an upright position during the morning with an electronic sphygmomanometer (model HEM-770A; Omron Health Care) by trained medical personnel. One of three cuff sizes, including adult standard, large, and thigh sized, were used depending on the circumference of the participant’s left arm. Serum creatinine was analyzed by isotope dilution mass spectrometry (IDMS)-traceable enzymatic method (Chen et al. 2014). Hypertension was defined as a systolic blood pressure (SBP) ≥ 140 mmHg or a diastolic blood pressure (DBP) ≥ 90 mmHg; overweight was defined as having a BMI ≥ 24 (kg/m^2^); diabetes mellitus was defined as having a fasting glucose ≥ 126 mg/dL; and hyperlipidemia was defined as having cholesterol ≥ 200 mg/dL. All of these classifications were classified based only on measurements taken at the time of the screening examination, without regard for prior history or treatment.

### Renal Function

We used the Taiwanese Chronic Kidney Disease Epidemiology Collaboration (CKD-EPI-Taiwan) equation to estimate glomerular filtration rate **(**eGFR): eGFR = 1.262 × {141 × *min*(*Scr*/κ,1)^α^ × *max*(*Scr*/κ,1)^1.209^ × 0.993^Age^ × 1.018[if female] × 1.159[if black]}^0.914^, where *Scr* is the serum creatinine, κ is 0.7 for females and 0.9 for males, α is –0.329 for females and –0.411 for males, *min* indicates the minimum of *Scr*/κ or 1, and *max* indicates the maximum of *Scr*/κ or 1 (Chen et al. 2014). The CKD-EPI-Taiwan equation performed better (when compared with inulin clearance) than the Modification of Diet in Renal Disease (MDRD) Study equation (Levey et al. 1999) or the Chronic Kidney Disease Epidemiology Collaboration (CKD-EPI) equation (Levey et al. 2009) in a validation study of Taiwanese adults. We classified CKD based on eGFR < 60 mL/min/1.73 m^2^, which represents a ≥ 50% reduction in normal kidney function (Levey et al. 2003).

### Particulate Matter Exposures

We estimated annual average concentrations of PM_2.5_, PM_2.5Absorbance_, PM_10_, and PM_Coarse_ (defined as PM_10_–PM_2.5_) at each participant’s residential address using an LUR model developed for the European Study of Cohorts for Air Pollution Effects (ESCAPE) project (http://www.escapeproject.eu/manuals) (Eeftens et al. 2012; Wang et al. 2013). To derive the model, we measured PM concentrations at 20 sampling sites (most of which were located in metropolitan Taipei) during three 14-day periods representing the intermediate (October–December 2009), cold (January–March 2010), and warm (June–August 2010) seasons. We used Harvard Impactors (Air Diagnostics and Engineering Inc.) to collect the particles, and a smoke stain reflectometer (model 43; Diffusion Systems Ltd.) to determine the PM_2.5Absorbance_ for the collected filters. We used ArcGIS, a geographic information system (GIS) (version 10.1; ESRI), to obtain land use and population data (buffers with radii of 100, 300, 500, 1,000, and 5,000 m); traffic data (buffers of 25, 50, 100, 300, 500, and 1,000 m); major roads (national highway, provincial highway, expressway, and city street); and elevated highways (roads established above ground level or highway ramp). We used supervised forward stepwise multiple regression to derive the final LUR models. Cross-validated R^2^ values were 0.74, 0.52, 0.91, and 0.92 for PM_10_, PM_Coarse_, PM_2.5_, and PM_2.5Absorbance_, respectively (Lee et al. 2015).

### Statistical Analyses

We used adjusted generalized linear regression models to estimate associations between interquartile range (IQR) increases in annual average PM exposures and eGFR and adjusted logistic regression models to estimate associations with prevalent CKD. Model covariates were age, fasting blood glucose, cholesterol, BMI, and distance to a major road (all modeled as continuous variables); sex; hypertension (yes if SBP ≥ 140 mmHg or DBP ≥ 90 mmHg, otherwise no); smoking (never, former, or current); alcohol consumption (never, former, seldom, or current); and education (uneducated, elementary or junior high, high school, college or graduate school). We used stratified analyses to examine whether associations with CKD differed according to sex, hypertension (yes or no), overweight (BMI ≥ 24 kg/m^2^ vs. < 24 kg/m^2^), diabetes mellitus (yes or no), smoking status (never, former, or current), distance to a major road (distance < 667.96 vs. ≥ 667.96 m), hyperlipidemia (cholesterol ≥ 200 mg/dL vs. < 200 mg/dL), age (age < 65 vs. ≥ 65), alcohol use (never, former, seldom, or current), or education (uneducated, elementary or junior high, high school, college or graduate school). Hierarchical models were used to control the confounding effect of the district. All of the analyses were performed using SAS software (version 9.3; SAS Institute).

## Results

Our study population consisted of adults with a mean age of 53.65 years, and the female to male ratio was approximately 2:1 (Table 1). Furthermore, 50.4% of the participants had a BMI higher than 24 kg/m^2^, and 33.2% were defined as having hypertension. Regarding health behaviors, 18.4% of patients had smoked, 36.6% had consumed alcohol, and 3.5% had chewed betel nut. The mean eGFR was 77.05 ± 13.18 mL/min/1.73 m^2^, and 10.3% (*n* = 2,226) were classified as having CKD based on eGFR < 60 mL/min/1.73 m^2^. The annual average concentration of PM_2.5_ was 26.64 μg/m^3^, which exceeded the guidelines of the World Health Organization (WHO 2006) in 2005 (10 μg/m^3^), the National Ambient Air Quality Standards (U.S. EPA 2016) in 2005 (12 μg/m^3^), and the standard of Taiwan in 2012 (15 μg/m^3^) (Taiwan EPA 2016). The annual average concentrations of PM_2.5Absorbance_, PM_10_, and PM_Coarse_ were 1.94 × 10^–5^/m, 49.48 μg/m^3^, and 23.13 μg/m^3^, respectively. The concentration of PM_10_ exceeded the annual mean value from the WHO guidelines in 2005 (20 μg/m^3^).

**Table 1:** Characteristics of the 21,656 participants in New Taipei City.

Variable	Mean ± SD or *n* (%)	Missing
Age (years)	53.65 ± 10.37	0
< 65	18,355 (84.8)
≥ 65	3,301 (15.2)
Sex, female	14,477 (66.9)	0
Body mass index (BMI) (kg/m^2^)	24.35 ± 3.51	44
Waist (cm)	79.21 ± 9.97	486
SBP (mmHg)	128.8 ± 20.16	46
DBP (mmHg)	81.8 ± 12.15	48
Fasting glucose (mg/dL)	100.61 ± 25.75	0
Cholesterol (mg/dL)	204.42 ± 36.54	2
Triglycerides (mg/dL)	118.73 ± 81.49	1
HDL (mg/dL)	68.96 ± 36.52	100
LDL (mg/dL)	113.06 ± 41.52	192
BUN (mg/dL)	13.48 ± 4.36	1
Creatinine (mg/dL)	0.84 ± 0.31	0
Hypertension, yes^*a*^	7,164 (33.2)	46
Overweight, yes^*b*^	10,902 (50.4)	44
Diabetes mellitus, yes^*c*^	1,550 (7.2)	0
Hyperlipidemia, yes^*d*^	11,405 (52.7)	2
Distance to major road (m)	667.96 ± 453.78	0
Away from major road^*e*^	9,729 (44.9)
Smoking		13
Never	17,664 (81.6)
Former	1,502 (6.9)
Current	2,477 (11.4)
Alcohol consumption		9
Never	13,716 (63.4)
Former	406 (1.9)
Seldom^*f*^	6,430 (29.7)
Current	1,095 (5.1)
Ever chew betel nut	763 (3.5)	22
Education level		166
Uneducated	1,523 (7.1)
Elementary or junior high school	9,179 (42.7)
High school	6,417 (29.9)
College or graduate school	4,371 (20.3)
Estimated glomerular filtration rate (eGFR)^*g*^	77.05 ± 13.18	0
Chronic kidney disease (CKD)^*h*^	2,226 (10.3)	0
1-year exposure		0
PM_2.5_ (μg/m^3^)	26.64 ± 5.01 (IQR = 5.67)
PM_2.5Absorbance_ (10^–5^/m)	1.94 ± 0.39 (IQR = 0.48)
PM_10_ (μg/m^3^)	49.48 ± 4.13 (IQR = 5.83)
PM_Coarse_ (μg/m^3^)	23.13 ± 3.68 (IQR = 6.59)
Note: HDL, high-density lipoprotein; IQR, interquartile range; LDL, low-density lipoprotein. ^***a***^Hypertension defined as systolic blood pressure (SBP) ≥ 140 mmHg or diastolic blood pressure (DBP) ≥ 90 mmHg. ^***b***^Overweight defined as BMI ≥ 24 (kg/m^2^). ^***c***^Diabetes mellitus defines as fasting glucose ≥ 126 mg/dL. ^***d***^Hyperlipidemia defined as cholesterol ≥ 200 mg/dL. ^***e***^Away from major road indicated as distance from major road greater than 667.96 m. ^***f***^Seldom indicated drink on special occasions (e.g, wedding or business entertainment). ^***g***^eGFR estimated by equation of CKD-EPI-Taiwan. ^***h***^CKD defined as eGFR ≤ 60 mL/min per 1.73 m^2^.		

eGFR was significantly lower in association with IQR increases in annual average PM_10_ [β = –0.69; 95% confidence interval (CI): –0.89, –0.48 for 5.83 μg/m^3^] and PM_Coarse_ (β = –1.06; 95% CI: –1.32, –0.81 for 6.59 μg/m^3^) based on a fully adjusted linear regression model (Table 2). The prevalence of CKD also was significantly higher in association with IQR increases in PM_10_ [odds ratio (OR) = 1.15; 95% CI: 1.07, 1.23] and PM_Coarse_ (OR = 1.26; 95% CI: 1.15, 1.38) based on a fully adjusted logistic regression model (Table 2). In contrast, neither eGFR nor CKD was significantly associated with IQR increases in PM_2.5_ or PM_2.5Absorbance_.

**Table 2:** Estimated associations of IQR increases in annual average PM exposures and eGFR or CKD (New Taipei City, *n* = 21,656).

Exposure	IQR	eGFR β (95% CI)	CKD^*a*^ OR (95% CI)
PM_2.5_	5.67 μg/m^3^	–0.09 (–0.25, 0.07)	1.03 (0.97, 1.09)
PM_2.5Absorbance_	0.48 × 10^–5^/m	0.02 (–0.16, 0.19)	1.03 (0.96, 1.09)
PM_10_	5.83 μg/m^3^	–0.69 (–0.89, –0.48)	1.15 (1.07, 1.23)
PM_Coarse_	6.59 μg/m^3^	–1.06 (–1.32, –0.81)	1.26 (1.15, 1.38)
Note: All models adjusted for age (years), sex, fasting glucose (mg/dL), cholesterol (mg/dL), hypertension (yes/no), BMI (kg/m^2^), distance to major road (m), smoking (never, former, current), alcohol consumption (never, former, seldom, current), and education (uneducated, elementary or junior high school, high school, college or graduate school). ^***a***^CKD defined as eGFR ≤ 60 mL/min per 1.73 m^2^.			

Overall, model estimates for associations of the PM exposures with eGFR and CKD were consistent with results from the main models when we adjusted for age only, and when we used hierarchical models to adjust for district of residence in addition to the other covariates included in the main model (see Tables S1 and S2).

**Figure 1:**
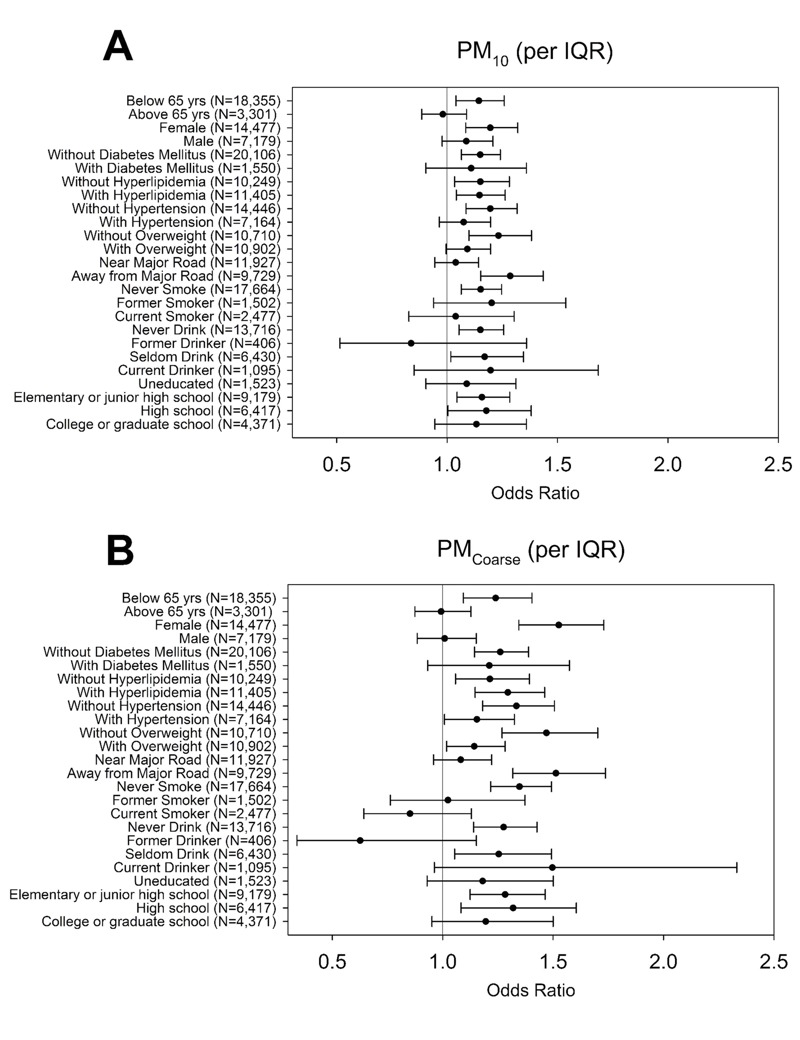
The odds of CKD for PM_10_ and PM_Coarse_ exposures stratified by age, sex, diabetes mellitus, hyperlipidemia, hypertension, overweight, distance to major road, smoking status, alcohol consumption, and education level among the 21,656 participants of New Taipei City.

**Figure 2:**
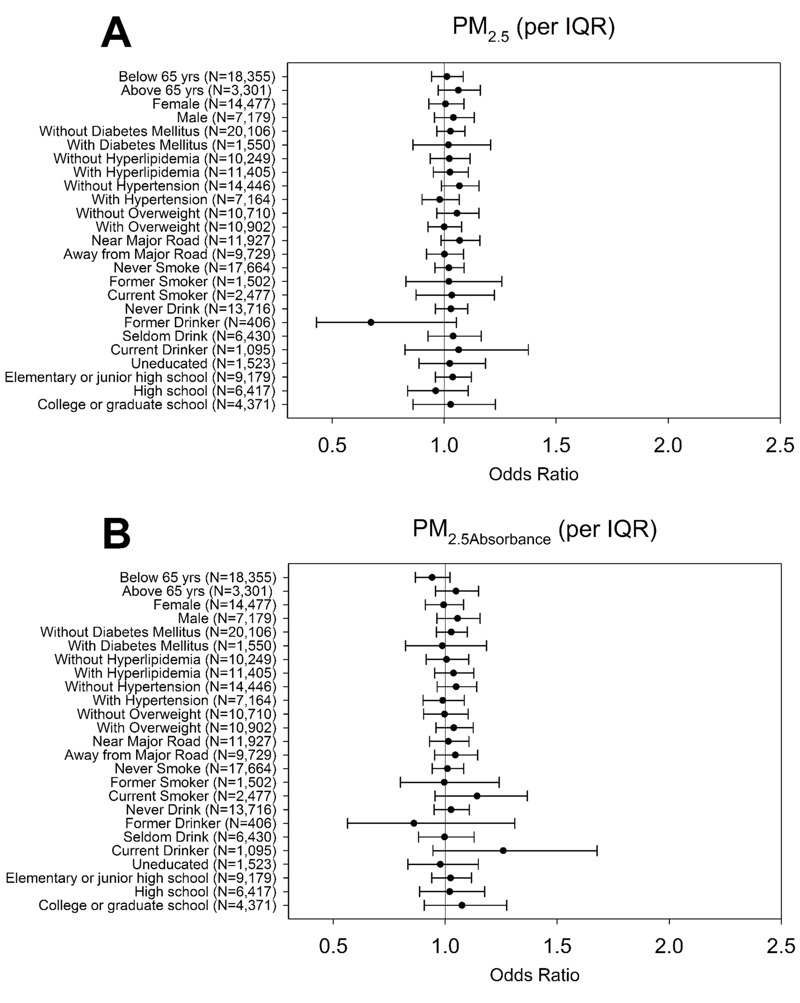
The odds of CKD for PM_2.5_ and PM_2.5Absorbance_ exposures stratified by age, sex, diabetes mellitus, hyperlipidemia, hypertension, overweight, distance to major road, smoking status, alcohol consumption, and education level among the 21,656 participants of New Taipei City.

Stratified analyses indicated that associations between CKD and PM_10_ were positive for those < 65 years of age, but null for older participants (Figure 1). In addition, positive associations between CKD and PM_10_ were stronger in females than males, in those without hypertension versus with hypertension, and in those who were normal weight versus overweight. PM_10_ also was more strongly associated with CKD among those who lived > 667.96 m from a major road compared with those who lived closer to a major road. Associations varied according to smoking status and alcohol use, but stratum-specific estimates were imprecise due to small numbers of cases within some of the groups. There was little variation in associations according to education or other factors evaluated. Patterns of associations between PM_Coarse_ and CKD according to population subgroups were similar to those for PM_10_, though the association appeared to be limited to females, as the association was null in males. Stratum-specific associations between CKD and PM_2.5_ and PM_2.5Absorbance_ were generally close to the null, without clear evidence of subgroup-specific effects (Figure 2). The same stratification analysis on eGFR showed the similar results (see Figures S1 and S2).

## Discussion

We successfully used LUR models to estimate within-city variation in PM exposures among 21,656 adult residents of New Taipei City, and the CKD-EPI-Taiwan equation, a modified version of the CKD-EPI equation (Chen et al. 2014), to estimate eGFR as a measure of renal function. We found that eGFR was inversely associated with average PM_10_ and PM_Coarse_ concentrations during the previous year. In addition, both exposures were associated with a higher prevalence of CKD, which was identified in 10% of our study population, consistent with the prevalence reported for Taiwan as a whole in 2003 (Kuo et al. 2007). Our findings are generally consistent with a recent longitudinal study that reported a negative association between long-term PM_2.5_ exposure and eGFR in 669 older American males (Mehta et al. 2016), though associations were specific to coarser fractions of PM in our study population. Living closer to a major road, an indicator of traffic related air pollution exposure, also was associated with lower eGFR in a study of 1,103 patients hospitalized for ischemic stroke (Lue et al. 2013). However, the urinary albumin/creatinine ratio, another indicator of renal function, was not associated with average 20-year exposure to PM_10_ or PM_2.5_ in 3,901 participants in the Multi-Ethnic Study of Atherosclerosis (MESA) cohort study (O’Neill et al. 2008).

Our findings also suggest that associations of CKD with PM exposures were stronger in females than males for PM_10_, and limited to females for PM_Coarse_. Previous studies have reported inconsistent findings for the effect of sex on the progression of CKD. A meta-analysis found that male patients with CKD displayed a faster rate of eGFR decline than their female counterparts (Neugarten et al. 2000). Conversely, another meta-analysis found that the adjusted hazard ratio for all-cause mortality was significantly higher in females than in males among those who had lower eGFR (Nitsch et al. 2013). An epidemiological study of CKD in Taiwan also showed that females had a higher incidence rate and risk of CKD compared with males (Kuo et al. 2007).

Due to a lack of information on socioeconomic or income data, we used education level as an indicator of socioeconomic level. A previous study found that a lower-socioeconomic status might be related to greater risk of CKD among white males (Merkin et al. 2005) and African Americans (Bruce et al. 2010). Although we adjusted for education as a proxy measure of socioeconomic status, we cannot rule out residual confounding by socioeconomic status in our study (Forastiere et al. 2007).

We also found stronger associations of PM_10_ and PM_Coarse_ with CKD among participants who were normal weight versus overweight, nonhypertension versus hypertension, and among those who lived > 667.96 m from a major road compared with those who lived closer to a major road. Previous studies have rarely addressed the population susceptibility of renal function to air pollution, but a few studies have observed an association between long-term PM exposure and impaired renal function among participants with comorbid diseases. In contrast to our findings, Mehta et al. 2016 reported that the negative association between PM_2.5_ and eGFR was stronger in nondiabetic than diabetic participants (interaction *p*-value 0.09), and stronger in those with high blood pressure versus normal blood pressure, and among obese versus nonobese participants (interaction *p*-values of 0.17 and 0.20, respectively) (Mehta et al. 2016). The protective effects of medication may be attributed to our findings of stronger PM-CKD associations among participants with healthy statuses or behaviors. Urinary 8-hydroxy-2´-deoxyguanosine (8-OHdG), a biomarker of reactive oxygen species (ROS)-induced DNA damage, was inversely associated with recent exposure to PM_2.5_ in 12 participants with hypertension, but positively associated with PM_2.5_ in 9 normotensive participants (Kim et al. 2009). Although hypertensive participants were significantly older, and were more likely to be smokers and to have diabetes or inflammatory lung disease than normotensive participants, 11 of 12 were taking antihypertensive medication that the authors suggested might have reduced DNA damage in response to PM_2.5_ through antioxidant effects (Kim et al. 2009). We did not have information on medication use in our study population, so were not able to determine whether medication use among participants with hypertension might have reduced their susceptibility to effects of PM on renal function.

Information from the Survey of Taiwan (https://olap.hpa.gov.tw/index.aspx) about the general population of New Taipei City > 30 years of age in 2009 suggests that our study population was more likely than the general population to be overweight (50% compared with 47%, respectively) and to have hypertension (33% compared with 22%, respectively), but were less likely to be current smokers (11% vs. 20%, respectively), alcohol consumers (37% vs. 55%, respectively), and betel nut chewers (3.5% vs. 8.3%, respectively).

There is limited research on the renal toxicity of inhaled ambient PM; however, it may partially share the pathway of PM-related cardiovascular toxicity. According to the American Heart Association’s scientific statement in 2010, airborne PM might lead to systematic oxidative stress, inflammation, thrombosis, vascular dysfunction, and atherosclerosis, which result in both macro- and micro-vascular damage (Brook et al. 2010). Because the kidney is highly vascularized and susceptible to atherosclerotic disease, PM might induce ischemic insults to the micro-vascular system of the kidney and might facilitate the progression toward chronic tubular damage (Tran et al. 2011). Previous studies also found that the risk of cardiovascular disease increased with a decline in eGFR (Go et al. 2004), and the risk of CKD was associated with inflammation (Muntner et al. 2004). An experimental study using a rat model of type 1 diabetes reported increased carotid intima thickness media and advanced glomerulosclerosis and tubular damage after 16 weeks of exposure to PM (average 13.30 μg/m^3^) compared with fresh air (Yan et al. 2014). The nephrotoxicity of smoking may also support our findings. Smoking has been recognized as an independent risk factor for CKD, and smoking might result in intraglomerular hypertension, vascular damage, or glomerulosclerosis via multiple complex interactions of nonhemodynamic (angiotensin II, transforming growth factor-β1, endothelin-1) and hemodynamic factors (Orth and Hallan 2008). Environmental (Galażyn-Sidorczuk et al. 2008) and occupational (El-Safty et al. 2004) exposures to cadmium and lead may affect the magnitude of kidney damage conferred by smoking. Pinto-Sietsma et al. found that participants who smoked more than 20 cigarettes/day might have a higher degree of eGFR decrease than others (Pinto-Sietsma et al. 2000).

It should be noted that we found a stronger association with PM_10_ and PM_Coarse_ than with PM_2.5_, which may be related to differences in the constituents of the particles that may influence their toxicity. An *in vitro* study found that the level of coarse particles was related to the endotoxin levels, which act as a pro-inflammatory indicator (Steenhof et al. 2011). Macrophages in the human lung were more easily stimulated by coarse particles than fine particles (Becker et al. 2002). The level of tumor necrosis factor-α was also more correlated with coarse particles than fine particles according to one study performed in Taipei (Huang et al. 2002). Cadmium, metallic mercury and lead exposures were reported to be related to kidney damage (Järup 2003). Cadmium exposure was reported to increase with the size of PM in Taipei (Wu et al. 2010). The size and composition of PM also appeared to have different effects on renal function, which warrants further study for confirmation.

There are several limitations of this investigation. First, we only estimated exposures to PM air pollution, and associations with gaseous pollutants and specific PM components should be evaluated in future studies. Second, although we have considered and controlled for individual characteristics and comorbid diseases, we did not have information on dietary habits or medication use, which may have confounded or modified exposure–outcome associations. Third, we did not adjust for proteinuria, which, along with low eGFR, is one of the most important risk factors for the progression of CKD (Astor et al. 2011; Tangri et al. 2011; Tonelli et al. 2011). Fourth, the fact that the stratum-specific OR for participants who were nondiabetic is significantly different from the null; whereas, the stratum-specific OR for participants who were diabetic is not, which is largely a consequence of the difference in the sample sizes. Additionally, eGFR was estimated based on a single serum creatinine measurement, which might have been influenced by recent diet, nutrition status, or inflammatory status (Branten et al. 2005; Quan et al. 1997). Finally, our findings, which were based on a population-based sample of adults ≥ 30 years of age, may not apply to specific population subgroups such as the elderly or those with specific comorbid conditions that might increase their susceptibility to renal disease as a consequence of PM exposure.

In conclusion, exposures to PM_10_ and PM_Coarse_ during the previous year were associated with lower eGFR and an increase in the prevalence of CKD among adult residents of Taipei City. Subgroup analyses suggested that these associations may be stronger *a*) among females than males, *b*) among younger versus older adults, *c*) among those who are normal weight versus overweight, *d*) among nonhypertensive versus hypertensive, and *e*) among those who live > 667.96 m from a major road. Longitudinal cohort studies are needed to confirm our findings.

## Supplemental Material

(420 KB) PDF
