# Supplementary material for: Associations between Long-Term Particulate Matter Exposure and Adult Renal Function in the Taipei Metropolis
Source: Environ Health Perspect. 2016 Oct 7;125(4):602–7. doi: 10.1289/EHP302 (PMC5381984; doi:10.1289/EHP302)

Environ Health Perspect DOI: 10.1289/EHP302
Advance Publication: Not Copyedited

Note to readers with disabilities: *EHP* strives to ensure that all journal content is accessible to all readers. However, some figures and Supplemental Material published in *EHP* articles may not conform to 508 standards due to the complexity of the information being presented. If you need assistance accessing journal content, please contact ehponline@niehs.nih.gov. Our staff will work with you to assess and meet your accessibility needs within 3 working days.

Supplemental Material

Associations between Long-Term Particulate Matter Exposure and Adult Renal Function in the Taipei Metropolis

Ya-Ru Yang, Yung-Ming Chen, Szu-Ying Chen, and Chang-Chuan Chan

Table of Contents

Table S1. Estimated associations [β (95% CI)] between IQR increases in annual average PM exposures and eGFR (New Taipei City, N = 21,656)

Table S2. Estimated associations [OR (95% CI)] between IQR increases in annual average PM exposures and CKD (New Taipei City, N = 21,656)

Figure S1. The associations of eGFR for $PM_{2.5}$ and $PM_{2.5Absorbance}$ exposures stratified by age, gender, diabetes mellitus, hyperlipidemia, hypertension, overweight, distance to major road, smoking status, alcohol consumption, and education level among the 21,656 subjects of New Taipei City.

Figure S2. The associations of eGFR for PM_{10} and PM_{Coarse} exposures stratified by age, gender, diabetes mellitus, hyperlipidemia, hypertension, overweight, distance to major road, smoking status, alcohol consumption, and education level among the 21,656 subjects of New Taipei City.

Table S1. Estimated associations [β (95% CI)] between IQR increases in annual average PM exposures and eGFR (New Taipei City, N = 21,656)

Exposure	IQR	Main model[a]	Age-adjusted[b]	Main + district[c]
$PM_{2.5}$	5.67 μg/m^3	–0.09 (–0.25, 0.07)	-0.03 (-0.19, 0.12)	–0.07 (–0.24, 0.09)
$PM_{2.5absorbance}$	0.48 × 10^{-5}/m	0.02 (–0.16, 0.19)	0.08 (-0.09, 0.26)	0.06 (–0.11, 0.24)
PM_{10}	5.83 μg/m^3	–0.69 (–0.89, –0.48)	-0.62 (-0.81, -0.42)	–0.53 (–0.79, –0.28)
PM_{coarse}	6.59 μg/m^3	–1.06 (–1.32, –0.81)	-1.00 (-1.25, -0.75)	–1.06 (–1.43, –0.68)

[a]Linear regression model adjusted for age (years), gender, fasting glucose (mg/dL), cholesterol (mg/dL), hypertension (yes/no), BMI (kg/m^2), distance to major road (m), smoking (never, former, current), alcohol consumption (never, former, seldom, current), and education (uneducated, elementary or junior high school, high school, college or graduate school).

[b]Linear regression model adjusted for age (years) only

[c]Hierarchical model adjusted for district (N = 6) and all main model covariates

Table S2. Estimated associations [OR (95% CI)] between IQR increases in annual average PM exposures and CKD (New Taipei City, N = 21,656)

Exposure	IQR	Main model[a]	Age-adjusted[b]	Main + district[c]
$PM_{2.5}$	5.67 μg/m^3	1.03 (0.97, 1.09)	1.02 (0.96, 1.08)	1.02 (0.96, 1.09)
$PM_{2.5absorbance}$	0.48 × 10^{-5}/m	1.03 (0.96, 1.09)	1.02 (0.96, 1.09)	0.98 (0.92, 1.05)
PM_{10}	5.83 μg/m^3	1.15 (1.07, 1.23)	1.13 (1.06, 1.21)	1.09 (0.99, 1.20)
PM_{coarse}	6.59 μg/m^3	1.26 (1.15, 1.38)	1.26 (1.16, 1.38)	1.16 (1.01, 1.34)

[a]Logistic regression model adjusted for age (years), gender, fasting glucose (mg/dL), cholesterol (mg/dL), hypertension (yes/no), BMI (kg/m^2), distance to major road (m), smoking (never, former, current), alcohol consumption (never, former, seldom, current), and education (uneducated, elementary or junior high school, high school, college or graduate school).

[b]Logistic regression model adjusted for age (years) only

[c]Hierarchical logistic regression model adjusted for district (N = 6) and all main model covariates

Figure S1. The associations of eGFR for $PM_{2.5}$ and $PM_{2.5Absorbance}$ exposures stratified by age, gender, diabetes mellitus, hyperlipidemia, hypertension, overweight, distance to major road, smoking status, alcohol consumption, and education level among the 21,656 subjects of New Taipei City.

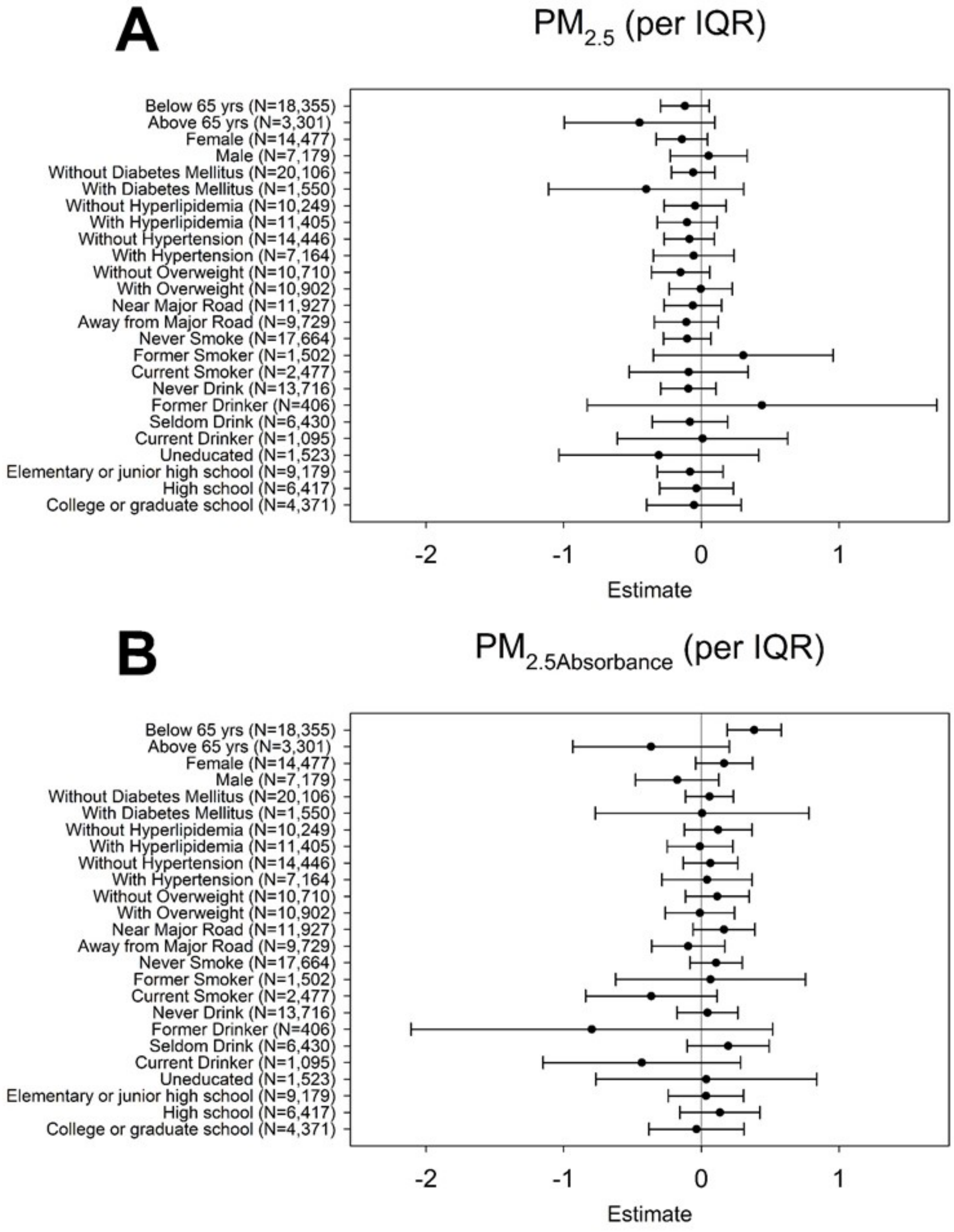

Figure S2. The associations of eGFR for PM_{10} and PM_{Coarse} exposures stratified by age, gender, diabetes mellitus, hyperlipidemia, hypertension, overweight, distance to major road, smoking status, alcohol consumption, and education level among the 21,656 subjects of New Taipei City.

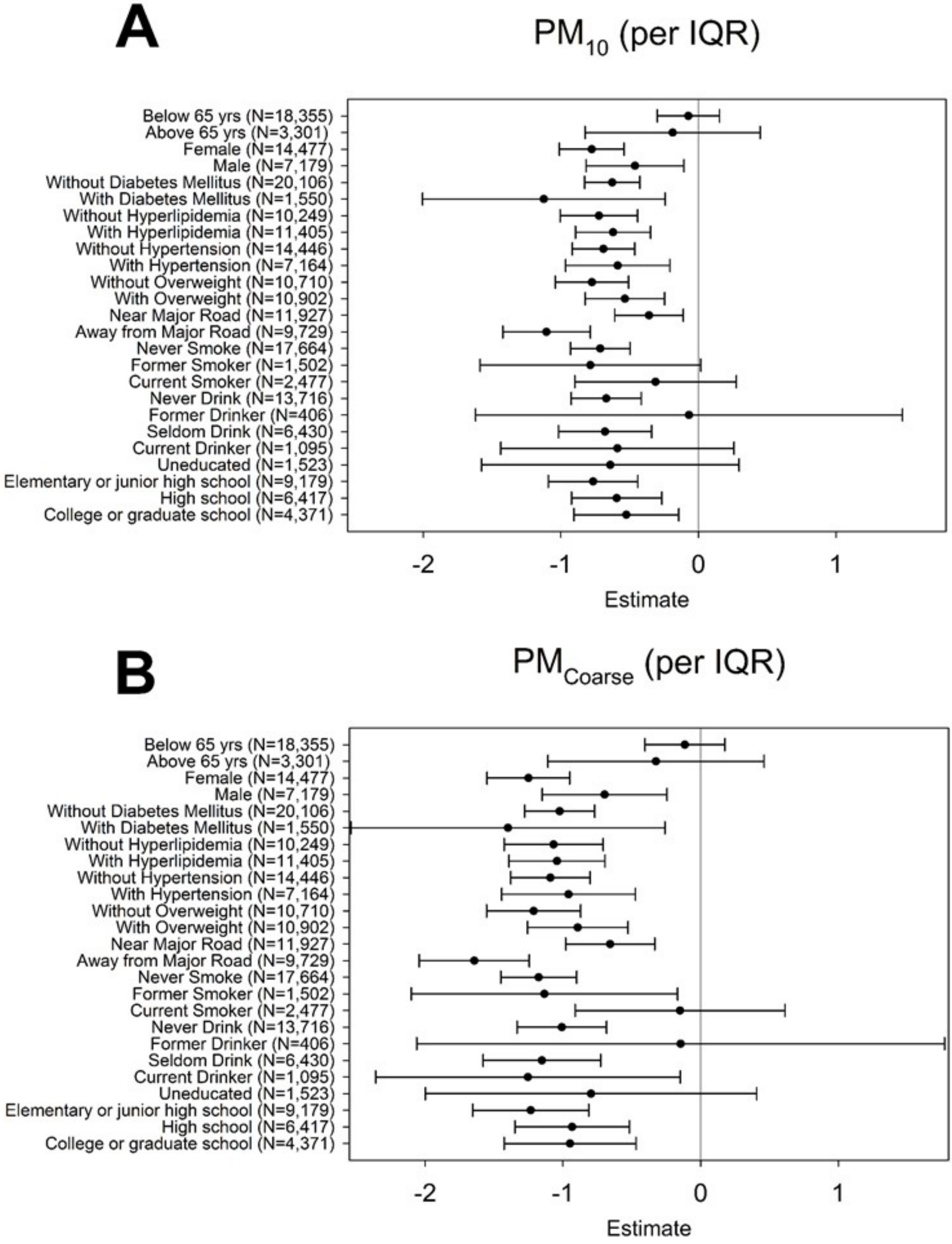

Supplement: (420 KB) PDF [file EHP302.s001.acco.pdf]
